# 

**Published:** 1895-06

**Authors:** 


### CONTENTS:

### SELECTIONS.

ARTICLE I.—Chapin A. Harris, M. D., D. D. S., By Samuel J. Cockerille, - - - - - - - - 49

" II.—Lycoperdon for Alveolar Hæmorrhage. By C. Brewster, L. D. S., - - - - - - - 53

" III.—Neuralgia. By Henry Blandy, L. D. S. - - 58

" IV.—Recurrence of Decay at the Cervix. By J. Foster Flagg, - - - - - - - - - 61

" V.—Mounting Logan Crowns. By J. W. Heckler, D. D. S., - - - - - - - - - 65

" VI.—Amalgams—Their Uses and Abuses. By A. C. Hewitt, M. D., - - - - - - - 66

" VII.—Treatment of Pulpless Teeth Having no Fstulous Opening. By Dr. C. N. Johnson, - - - 69

### COMMUNICATED.

The American Dental Association, - - - - - - 73
Illinois State Dental Society, - - - - - - - 74
National Association of Dental Faculties, - - - - 74
The National Association of Dental Examiners. - - - 75
New Jersey State Dental Society, - - - - - - 75

### EDITORIAL, ETC.

Chapin A. Harris, A. M., M. D., D. D. S., - - - 76
Professional Habits, - - - - - - - - 79
English Dentists, - - - - - - - - - - 80
University of Maryland, Dental Department. - - - 81
The Baltimore College of Dental Surgery, - - - - - 82
University of Buffalo, Dental Department, - - - - - 83

### BIBLIOGRAPHICAL.

The Anatomy and Pathology of the Teeth, - - - - - 84

Taking Impressions of the Mouth - - - - - - 86

Manual of Chemistry, - - - - - - - - - 86

General Surgery and Pathology for Dentists, - - - - 87

Catching's Compendium of Practical Dentistry for 1894, - - 88

Marchand on Peroxide of Hydrogen, Glycozone and Hydrozone. 88

### MONTHLY SUMMARY.

Rational Therapeutics of Cholera Infantum, - - - - 88

Filling Teeth, - - - - - - - - - - - 91

Horace Wells Anniversary Proceedings, - - - - - 91

The First Permanent Molar, - - - - - - - 92

Very Soft Teeth, - - - - - - - - - - 92

A Liquid which will Mix Almost any Cement Filling, - - 92

Water Cure, - - - - - - - - - - - 93

White Fillings, - - - - - - - - - - 93

Cinnamon Drink for Fever Patients. - - - - - 93

English Dental Examinations, - - - - - - 94

To Remove Remnants of Putrescent Tissue in Root-canals, - 94

Rubber, - - - - - - - - - - - - 84

A Set of Teeth as a Detective, - - - - - - - 95

To Bleace a Tooth, - - - - - - - - - 95

Sheep with Golden Teeth, - - - - - - - 95

Root-canal Filling, - - - - - - - - - 96

Facial Paralysis, - - - - - - - - - - 96

